# Supplementary figures and images for: Cell-type-specific firing patterns in a V1 cortical column model depend on feedforward and feedback-driven states
Source: PLoS Comput Biol. 2025 Apr 23;21(4):e1012036. doi: 10.1371/journal.pcbi.1012036 (PMC12017539; doi:10.1371/journal.pcbi.1012036)

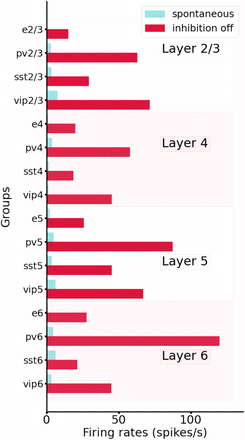

Supplement: S1 Fig — All groups substantially elevated their firing rates, particularly those in deep layers. (TIF) [file pcbi.1012036.s001.tif]

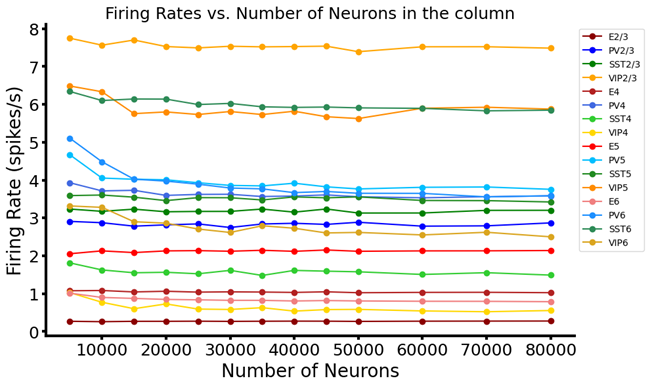

Supplement: S2 Fig — We show that, with proper scaling of the weights (see Methods), results for 5k,10k up to 80k neurons lead to very similar results. (TIF) [file pcbi.1012036.s002.tif]

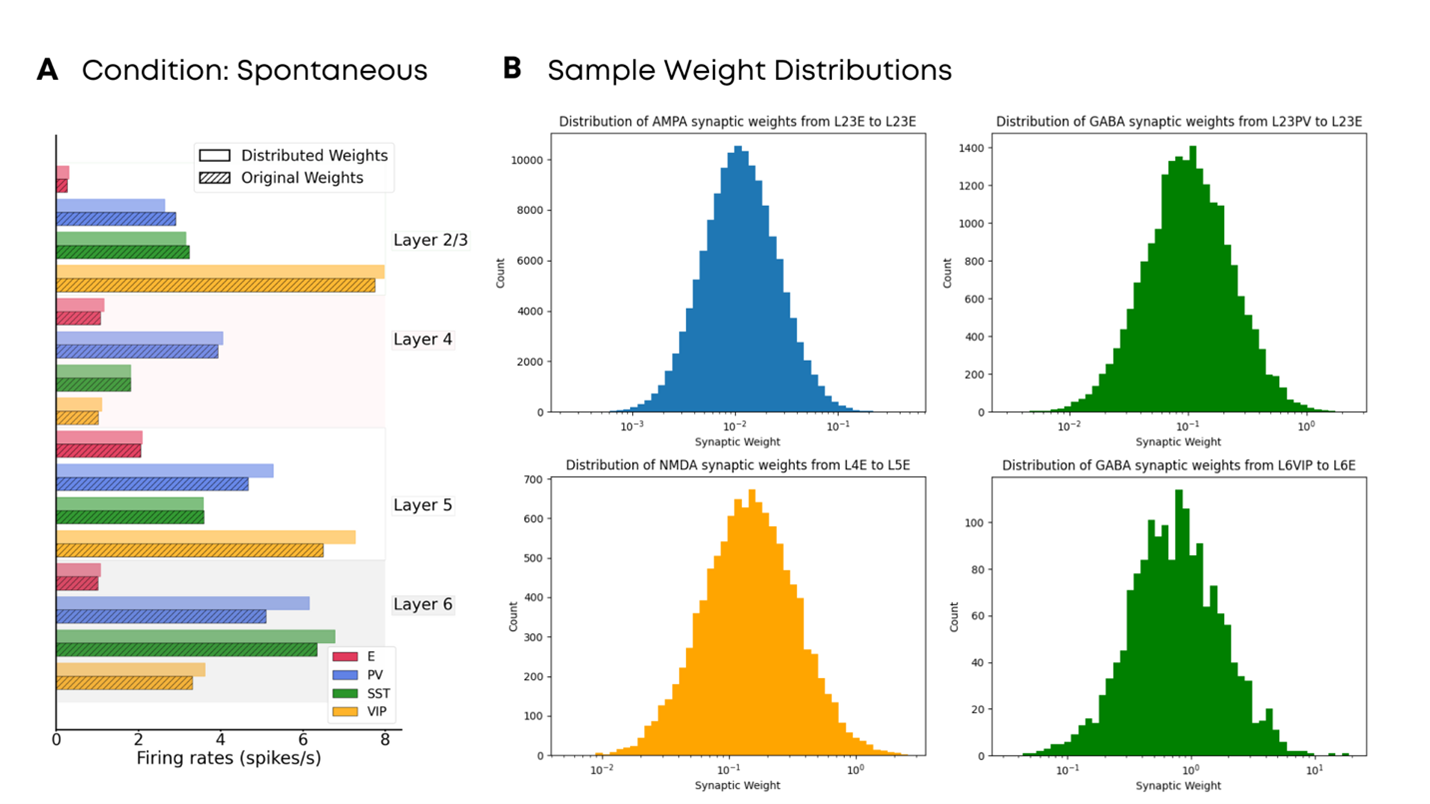

Supplement: S3 Fig — A) Comparison between the mean firing rates for all layers and cell types of the original model shown in Figs 1 and 2 and a model in which values for each individual synaptic weight are chosen from a lognormal distribution, as reported experimentally. The mean of the distribution corresponds to the original value of the projection in the original model, and the standard deviation is set equal to the mean. B) Examples for the resulting distribution of synaptic weights in the modified model, for several synapse and cell types as indicated in the panel titles. For most cases, the distribution of weights spans about two orders of magnitude, in agreement with experimental observations. (TIF) [file pcbi.1012036.s003.tif]

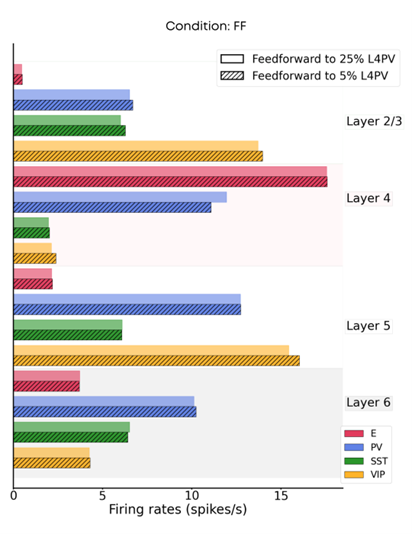

Supplement: S4 Fig — The figure compares the mean firing rates across layers and cell types for the original model shown in Figs 1 and 2, and a modified model in which the feedforward input arrives at 25% of the PV cells in layer 4, instead of just 5% as in the original model. For the modified model, input weights to layer 4 PV cells were scaled down by a factor of 0.25 to compensate for the additional inhibition. (TIF) [file pcbi.1012036.s004.tif]

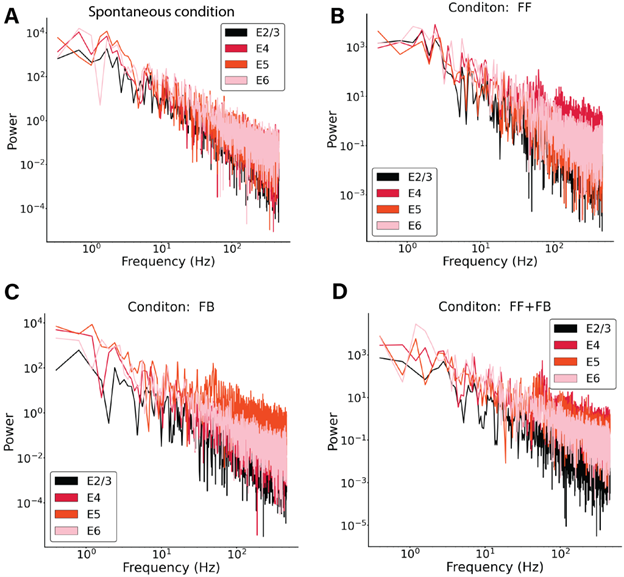

Supplement: S5 Fig — A) Spontaneous condition. B) Feedforward input of 150pA injected in 25% excitatory cells and 5% Pv cells in layer 4. C) Feedback input of 150pA injected in 25% excitatory cells and 5% Pv cells in layer 5. D) Feedback input of 150pA injected in 25% excitatory cells and 5% Pv cells in layer 5 and layer 4. In all conditions no signs of oscillatory activity are shown. The subset of cells receiving the input shows some level of synchronisation, which is reflected in a small peak in their corresponding power spectrum. (TIF) [file pcbi.1012036.s005.tif]

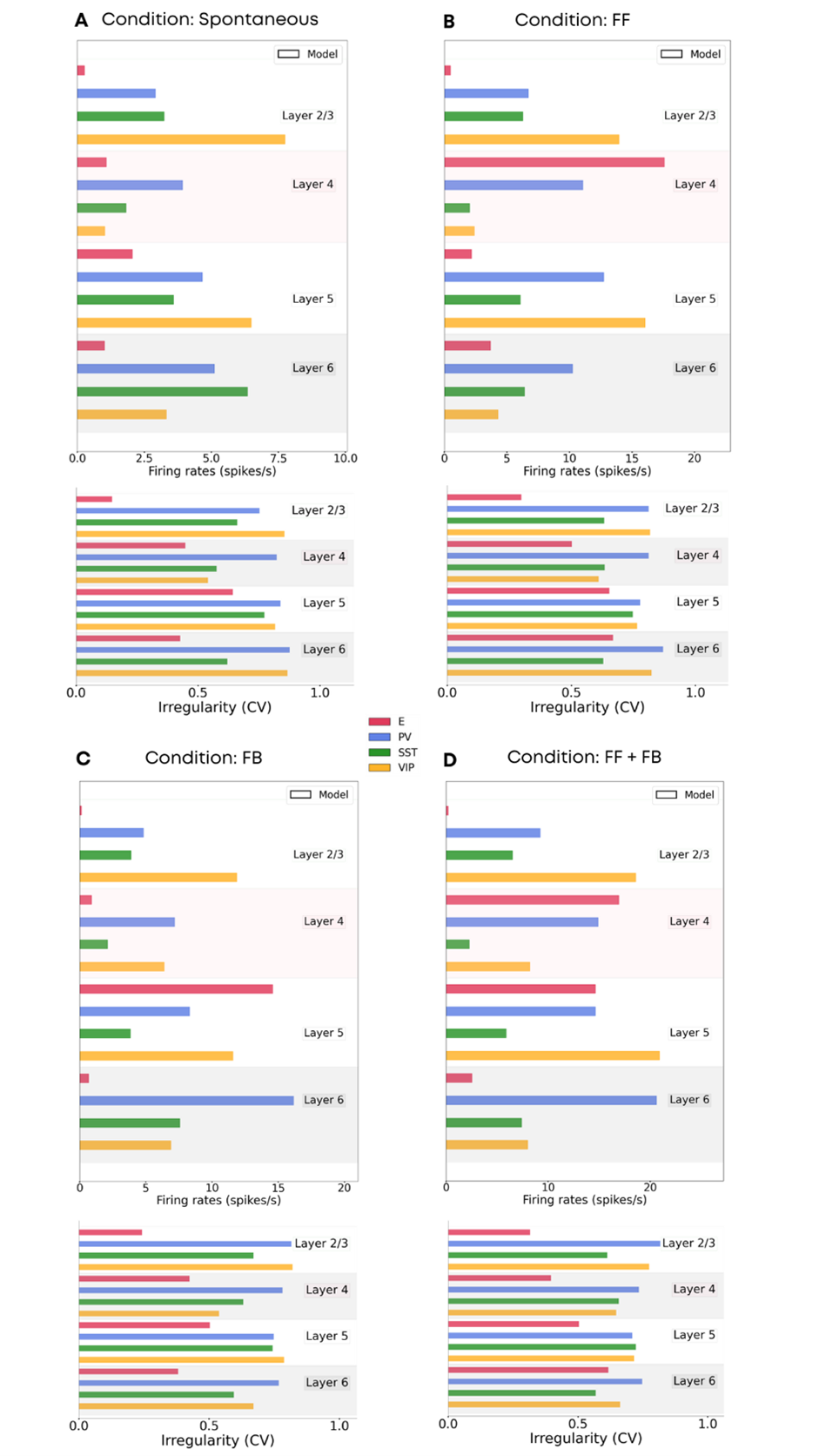

Supplement: S6 Fig — A) Spontaneous condition B) Feedforward input of 150pA injected in 25% excitatory cells and 5% Pv cells in layer 4. C) Feedback input of 150pA injected in 25% excitatory cells and 5% Pv cells in layer 5. D) Feedback input of 150pA injected in 25% excitatory cells and 5% Pv cells in layer 5 and layer 4. In all conditions most cells have a CV > 0.5 showing no synchrony. In A) and C) excitatory cells in layer 2/3 have a low CV, probably due to the fact that the firing rate activity is very low, therefore the CV is harder to properly evaluate. (TIF) [file pcbi.1012036.s006.tif]

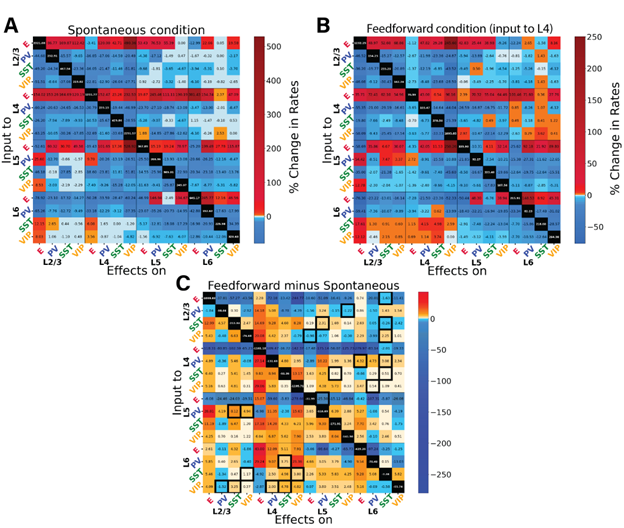

Supplement: S7 Fig — Panels (A), (B) and (C) provide additional information on the data presented in Fig 3. (A) Matrix of input-output relationships of the network in the spontaneous state. We delivered excitatory input to one population (Y-axis) and observed its effect on others (X-axis), repeating this for all 16 populations to construct the matrix. We stimulated each subpopulation with a 30 pA DC current and monitored the resultant firing rate changes in all other subpopulations. This matrix shows the exact percentage changes of the firing rates compared to the situation pre- perturbative injection. The diagonal (change of firing rate of the perturbed cells) are removed from the colour code and set to black. (B) Displays the response matrix for the feedforward-driven state, wherein excitatory input is provided to a subset of L4 pyramidal cells and PV cells (Input of 150pA to 25% of E4 and 5% of PV4). (C) Matrix illustrating the difference in percentage change between the two conditions (i.e., if in the spontaneous case the percentage change is + 50% and in the FF is + 30%, the difference matrix will show -20%). The black contour square indicates when a change in sign between the two matrices occurred. (TIF) [file pcbi.1012036.s007.tif]

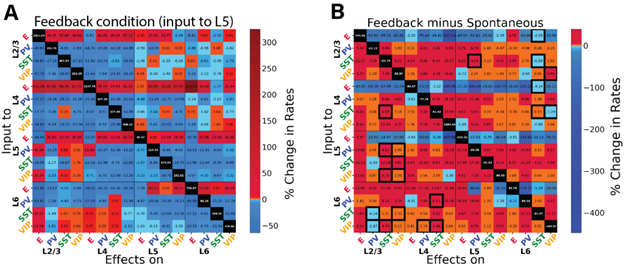

Supplement: S8 Fig — Panels (A) and (B) provide additional information on the data presented in Fig 4. (A) Perturbational matrix of input-output relationships within the network. With feedback input (150pA input to 25% pyramidal cells and 5% PV cells in layer 5) applied, we administered input to one population (indicated on the Y-axis) and observed the effects on the others (X-axis). This process was repeated for all 16 populations to compile the matrix. We stimulated each subpopulation with a 30 pA DC current and monitored the resultant firing rate changes in all other populations. The matrix shows the exact percentage changes of the firing rates compared to the situation pre- perturbation injection. (B) Comparative matrix between the feedback and spontaneous conditions. (TIF) [file pcbi.1012036.s008.tif]

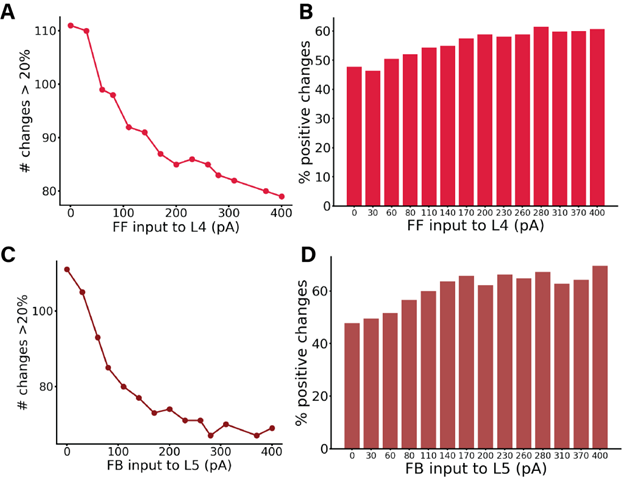

Supplement: S9 Fig — (A) displays the number of changes >20% (or < -20%) in the corresponding perturbation matrix. We conducted perturbation analyses for 14 different network conditions, defined by varying feedforward (FF) input to layer 4, resulting in a 16x16 matrix for each cell group, although these matrices are not displayed here. Each condition varied the input strength to excitatory neurons in layer 4, with values on the X-axis ranging from 0 to 400 pA. The Y-axis represents the number of alterations >20% (or < -20%) in the respective matrix (the sum of red and blue squares). Increased input to layer 4 results in fewer perturbation-induced changes in the firing rates of other populations. (B) shows percentages of positive changes in firing rates elicited by all possible perturbations in each network state, computed from the total number of changes presented in A. The complementary percentages in the panel also provide data for the negative changes. (C) displays the number of changes >20% (or < -20%) in the corresponding perturbation matrix for 14 different network conditions, defined by varying feedback (FB) input to layer 5, resulting in a 16x16 matrix for each cell group, although these matrices are not displayed here. Each condition varied the input strength to excitatory neurons in layer 5, with values on the X-axis ranging from 0 to 400 pA. The Y-axis represents the number of substantial alterations >20% (or < -20%) in the respective matrix (the sum of red and blue squares). Increased input to layer 5 results in fewer perturbation-induced changes in the firing rates of other populations. (D) presents percentages of positive changes in firing rates elicited by all possible perturbations in each network state, computed from the total number of changes shown in C. The complementary percentages in the panel also provide data for the negative changes. (TIF) [file pcbi.1012036.s009.tif]

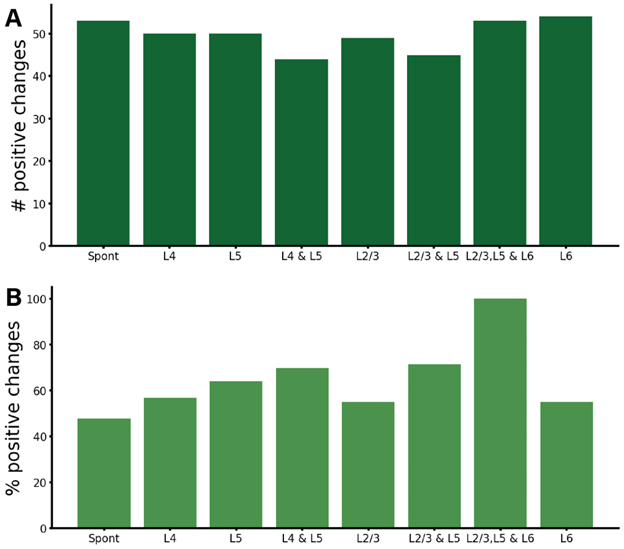

Supplement: S10 Fig — >20% (or < -20%) (A) and percentages (B) for positive changes in firing rates elicited by all possible perturbations in the spontaneous state (Spont), feedforward-driven state (L4), feedforward-feedback combination (L4 & L5), and feedback-driven state in all its five possible configurations (i.e., targeting subset of pyramidal and PV in layer 5 (L5),subset of pyramidal and PV cells in layer 2/3 (L23), subset of pyramidal and PB cells in layer 2/3 and 5 (L2/3 & L5), subset of pyramidal and PV cells in layer 2/3,5 and 6 (L2/3, L5 & L6), and subset of pyramidal and PV cells in layer 6(L6)). The complementary numbers for the bottom panel also provide the data for the negative changes. This figure provides extra analysis on the data presented in Figs 3A, 3B, 4A, 5B and 6C. (TIF) [file pcbi.1012036.s010.tif]

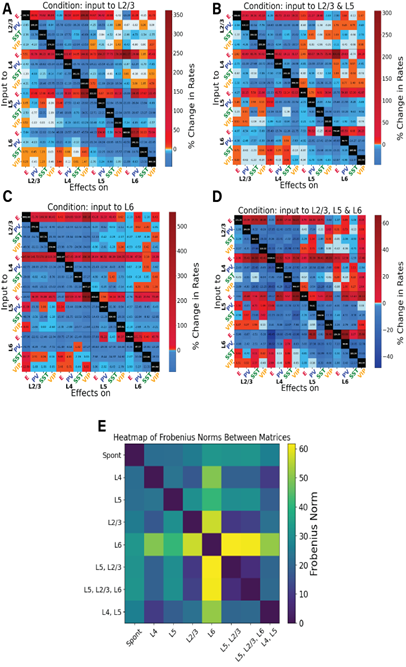

Supplement: S11 Fig — Panels (A), (B), (C), (D) provide additional information on the data presented in Fig 5. (A)-(D) Perturbation Input-output matrices for four distinct feedback states: A) Input to subset of E and PV neurons in layer 2/3. B) Input to subset of E and PV neurons in layer 2/3 and layer 5. C) Input to subset of E and PV neurons in layer 6. D) Input to subset of E and PV neurons in layer 2/3, 5 and 6. Within each condition, we delivered a 30 pA DC current to one population (Y-axis) and observed the resultant effects on the others (X-axis). We replicated this procedure for all 16 populations to derive each matrix. The matrices show the exact percentage changes of the firing rates compared to the situation pre- perturbation injection. (E) To quantify the differences between the conditions we used the Frobenius norm: we compute the pairwise distances between all the matrices to see which conditions are closer to each other. (TIF) [file pcbi.1012036.s011.tif]

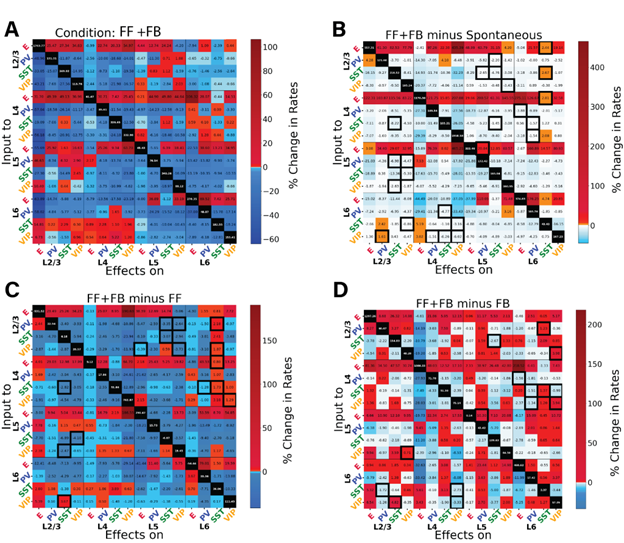

Supplement: S12 Fig — Panels (A), (B), (C), (D) provide additional information on the data presented in Fig 6. (A) Matrix of input-output relationships of the network. When both inputs to L4 and L5 are present, we delivered a 30 pA DC current to one population (Y-axis) and observed the resultant effects on the others (X-axis). We repeated this procedure for the 16 populations to obtain the matrix. The matrices show the exact percentage changes of the firing rates compared to the situation pre- perturbation injection. (B) Matrix showing the difference between the feedback & feedforward situation and the spontaneous condition. (C) Matrix showing the difference between the feedback & feedforward situation and the feedforward only condition. (D) Matrix showing the difference between the feedback & feedforward situation and the feedback only condition. The black contour square indicates when a change in sign between the two matrices occurred. (TIF) [file pcbi.1012036.s012.tif]

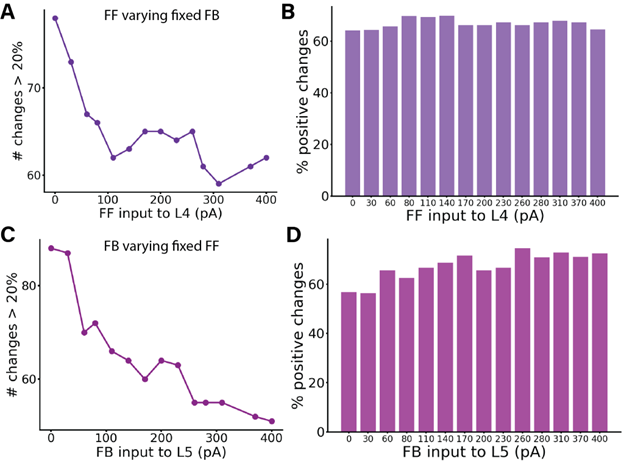

Supplement: S13 Fig — C-D) Varying FB input while keeping FB constant. (A) displays the number of changes >20% (or < -20%) in the corresponding perturbation matrix. Perturbation analysis was conducted for 14 different network conditions, defined by varying feedforward (FF) input to layer 4 while keeping feedback (FB) input to a subset of pyramidal cells and PV cells in layer 5 constant at 150 pA, resulting in a 16x16 matrix for each cell group, although these matrices are not displayed here. Each condition varied the input strength to neurons in layer 4 (25% E4 and 5% PV4), with values on the X-axis ranging from 0 to 400 pA. The Y-axis represents the number of alterations >20% (or < -20%) in the respective matrix (the sum of red and blue squares). Increased input to layer 4 results in fewer perturbation-induced changes in the firing rates of other populations. (B) presents percentages of positive changes in firing rates elicited by all possible perturbations in each network state, computed from the total number of changes shown in A. The complementary percentages in the panel also provide data for the negative changes. (C) illustrates the number of changes >20% (or < -20%) in the corresponding perturbation matrix. Perturbation analysis was conducted for 14 different network conditions, defined by varying feedback (FB) input to layer 5, and resulting in a 16x16 matrix for each cell group, though these matrices are not displayed here. Each condition varied the input strength to a subset of excitatory and PV neurons in layer 5, with values on the X-axis ranging from 0 to 400 pA. The Y-axis represents the number of t alterations >20% (or < -20%) in the respective matrix (the sum of red and blue squares). Increased input to layer 5 results in fewer perturbation-induced changes in the firing rates of other populations. (D) shows percentages of positive changes in firing rates elicited by all possible perturbations in each network state. The percentage is computed from the total number of chang [file pcbi.1012036.s013.tif]

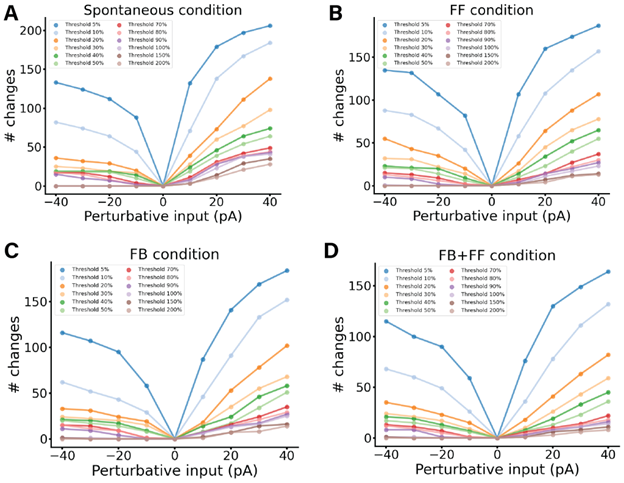

Supplement: S14 Fig — A) Spontaneous condition. The perturbation values used are [-40, -30, -20, -10, 10, 20, 30, 40] pA, for each value a perturbation analysis is carried out and a perturbation matrix is created. As usual to construct the matrix we delivered perturbative input to one population and observed its effect on others, repeating this for all 16 populations. We stimulated each subpopulation with a DC current of a given strength and monitored the resultant firing rate changes in all other subpopulations. Then for each matrix we counted the number of cells which changed their firing rate by more than the chosen threshold. Therefore we were able to draw the coloured lines in the plot. The threshold chosen for the figure are: 5%, 10%, 20%, 30%, 40%, 50%, 70%,80%, 90%, 100%, 150%, 200% indicating an increase or decrease of the firing rate of that quantity. B) Same analysis for Feedforward-driven state, wherein input is provided to a subset of L4 pyramidal cells and PV cells. C) Same analysis for Feedback-driven state, wherein input is provided to a subset of L5 pyramidal cells and PV cells. D) Same analysis for Feedforward and Feedback-driven state, wherein input is provided to a subset of L5 and L4 pyramidal cells and PV cells. For all condition the more perturbative input is injected the more the cells are changing their firing rate. (TIF) [file pcbi.1012036.s014.tif]

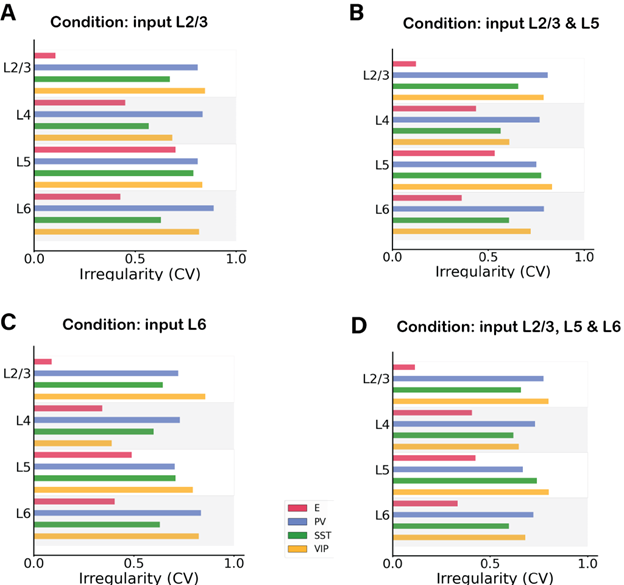

Supplement: S15 Fig — A) Condition: Feedback input to subset of E and PV neurons in layer 2/3. B) Condition: Input to subset of E and PV neurons in layer 2/3 and layer 5. C) Condition: Input to subset of E and PV neurons in layer 6. D) Condition: Input to subset of E and PV excitatory neurons in layer 2/3, 5 and 6. In all conditions most cells have a CV > 0.5 or around 0.5 showing no synchrony. Excitatory cells in layer 2/3 have a low CV, caused by the firing rate activity being very low for those cells, and therefore making the CV harder to properly evaluate. (TIF) [file pcbi.1012036.s015.tif]

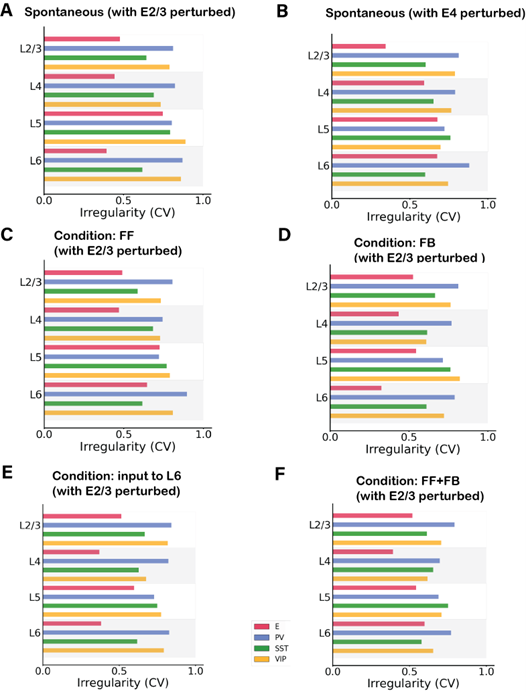

Supplement: S16 Fig — A) Condition: Spontaneous with perturbation of 30pA in excitatory neurons in layer 2/3. B) Condition: Spontaneous with perturbation of 30pA in excitatory neurons in layer 4. C) Condition: Feedforward input of 150pA injected in 25% excitatory cells and 5% PV cells in layer 4 and perturbative input to excitatory cells in layer 2/3. D) Condition: Feedback input of 150pA injected in 25% excitatory cells and 5% PV cells in layer 5 and perturbative input to excitatory cells in layer 2/3. E) Feedback input of 150pA injected in 25% excitatory cells and 5% PV cells in layer 6 and perturbative input to excitatory cells in layer 2/3. F) Condition: Feedforward and Feedback input combined, 150pA injected in 25% excitatory cells and 5% PV cells in layer 4 and 5 and perturbative input to excitatory cells in layer 2/3. In all conditions most cells have a CV > 0.5 or around 0.5 showing no synchrony. (TIF) [file pcbi.1012036.s016.tif]
