# Supplementary material for: Cell-type-specific firing patterns in a V1 cortical column model depend on feedforward and feedback-driven states
Source: PLoS Comput Biol. 2025 Apr 23;21(4):e1012036. doi: 10.1371/journal.pcbi.1012036 (PMC12017539; doi:10.1371/journal.pcbi.1012036)
Supplement: S1 Table — Values represent the Poisson generator rates υbg. (DOCX) [file pcbi.1012036.s017.docx]

*Table 1:*

| *Rate of Poisson generator υbg (Hz)* | *E* | *PV* | *SST* | *VIP* |
| --- | --- | --- | --- | --- |
| *L1* |  |  |  | *650* |
| *L2/3* | *930* | *1460* | *870* | *1405* |
| *L4* | *890* | *1984* | *2105* | *240* |
| *L5* | *4740* | *930* | *530* | *870* |
| *L6* | *1770* | *1170* | *885* | *1620* |
