# Supplementary material for: Cell-type-specific firing patterns in a V1 cortical column model depend on feedforward and feedback-driven states
Source: PLoS Comput Biol. 2025 Apr 23;21(4):e1012036. doi: 10.1371/journal.pcbi.1012036 (PMC12017539; doi:10.1371/journal.pcbi.1012036)
Supplement: S2 Table — Ntot is the total number of cells in the column and can be defined arbitrarily for a simulation. The number of cells in each layer will scale accordingly [23]. We used Ntot = 5000 for simulations. (DOCX) [file pcbi.1012036.s018.docx]

*Table 2:*

|  | *Number of neurons* |
| --- | --- |
| *L1* | *0.0192574218*Ntot* |
| *L2/3* | *0.291088453*Ntot* |
| *L4* | *0.237625904*Ntot* |
| *L5* | *0.17425693*Ntot* |
| *L6* | *0.297031276*Ntot* |
