# Supplementary material for: Cell-type-specific firing patterns in a V1 cortical column model depend on feedforward and feedback-driven states
Source: PLoS Comput Biol. 2025 Apr 23;21(4):e1012036. doi: 10.1371/journal.pcbi.1012036 (PMC12017539; doi:10.1371/journal.pcbi.1012036)
Supplement: S3 Table — In each layer, the inhibitory cells represent 15% of the total number of neurons for that layer [23]. (DOCX) [file pcbi.1012036.s019.docx]

*Table 3:*

| *Percentage (%)* | *PV* | *SST* | *VIP* |
| --- | --- | --- | --- |
| *L2/3* | *0.295918* | *0.214286* | *0.489796* |
| *L4* | *0.552381* | *0.295238* | *0.152381* |
| *L5* | *0.485714* | *0.428571* | *0.085714* |
| *L6* | *0.458333* | *0.458333* | *0.083333* |
