# Supplementary material for: Cell-type-specific firing patterns in a V1 cortical column model depend on feedforward and feedback-driven states
Source: PLoS Comput Biol. 2025 Apr 23;21(4):e1012036. doi: 10.1371/journal.pcbi.1012036 (PMC12017539; doi:10.1371/journal.pcbi.1012036)
Supplement: S4 Table — (DOCX) [file pcbi.1012036.s020.docx]

*Table 4:*

| *Number of neurons* | *E* | *PV* | *SST* | *VIP* |
| --- | --- | --- | --- | --- |
| *L1* |  |  |  | *96* |
| *L2/3* | *1236* | *65* | *47* | *107* |
| *L4* | *1010* | *98* | *53* | *27* |
| *L5* | *741* | *63* | *56* | *11* |
| *L6* | *1263* | *102* | *102* | *19* |
