# Supplementary material for: Cell-type-specific firing patterns in a V1 cortical column model depend on feedforward and feedback-driven states
Source: PLoS Comput Biol. 2025 Apr 23;21(4):e1012036. doi: 10.1371/journal.pcbi.1012036 (PMC12017539; doi:10.1371/journal.pcbi.1012036)
Supplement: S5 Table — The values are taken from the Allen database [23]. The same values are used for all simulations. (DOCX) [file pcbi.1012036.s021.docx]

*Table 5:*

| *Cm (pF)* | *E* | *PV* | *SST* | *VIP* |
| --- | --- | --- | --- | --- |
| *L1* |  |  |  | *37.11* |
| *L2/3* | *123.41* | *70.95* | *82.34* | *41.23* |
| *L4* | *80.16* | *81.21* | *132.86* | *40.3* |
| *L5* | *149.43* | *70.9* | *52.32* | *59.29* |
| *L6* | *99.96* | *49.65* | *96.09* | *65.87* |
