# Supplementary material for: Cell-type-specific firing patterns in a V1 cortical column model depend on feedforward and feedback-driven states
Source: PLoS Comput Biol. 2025 Apr 23;21(4):e1012036. doi: 10.1371/journal.pcbi.1012036 (PMC12017539; doi:10.1371/journal.pcbi.1012036)
Supplement: S6 Table — (DOCX) [file pcbi.1012036.s022.docx]

*Table 6:*

| *gL (nS)* | *E* | *PV* | *SST* | *VIP* |
| --- | --- | --- | --- | --- |
| *L1* |  |  |  | *4.07* |
| *L2/3* | *2.47* | *9.49* | *3.17* | *6.4* |
| *L4* | *5.16* | *9.19* | *7.96* | *1.87* |
| *L5* | *16.66* | *5.21* | *3.43* | *6.52* |
| *L6* | *5.88* | *6.86* | *2.99* | *6.09* |
|  |  |  |  |  |
