# Supplementary material for: Cell-type-specific firing patterns in a V1 cortical column model depend on feedforward and feedback-driven states
Source: PLoS Comput Biol. 2025 Apr 23;21(4):e1012036. doi: 10.1371/journal.pcbi.1012036 (PMC12017539; doi:10.1371/journal.pcbi.1012036)
Supplement: S7 Table — (DOCX) [file pcbi.1012036.s023.docx]

*Table 7:*

| *τref (ms)* | *E* | *PV* | *SST* | *VIP* |
| --- | --- | --- | --- | --- |
| *L1* |  |  |  | *3.5* |
| *L2/3* | *3* | *1.26* | *1.85* | *2.75* |
| *L4* | *4.4* | *1.5* | *2.2* | *2.4* |
| *L5* | *4.25* | *1.85* | *1.9* | *2.55* |
| *L6* | *3.3* | *1.65* | *2.1* | *2.85* |
