# Supplementary material for: Cell-type-specific firing patterns in a V1 cortical column model depend on feedforward and feedback-driven states
Source: PLoS Comput Biol. 2025 Apr 23;21(4):e1012036. doi: 10.1371/journal.pcbi.1012036 (PMC12017539; doi:10.1371/journal.pcbi.1012036)
Supplement: S8 Table — (DOCX) [file pcbi.1012036.s024.docx]

*Table 8:*

| *Vrest (mV)* | *E* | *PV* | *SST* | *VIP* |
| --- | --- | --- | --- | --- |
| *L1* |  |  |  | *-65.5* |
| *L2/3* | *-80.97* | *-82.35* | *-69.16* | *-67.94* |
| *L4* | *-72.53* | *-70.45* | *-74.2* | *-63.14* |
| *L5* | *-68.28* | *-77.5* | *-70.01* | *-72.00* |
| *L6* | *-77.5* | *-76.42* | *-62.99* | *-78.85* |
