# Supplementary material for: Cell-type-specific firing patterns in a V1 cortical column model depend on feedforward and feedback-driven states
Source: PLoS Comput Biol. 2025 Apr 23;21(4):e1012036. doi: 10.1371/journal.pcbi.1012036 (PMC12017539; doi:10.1371/journal.pcbi.1012036)
Supplement: S9 Table — (DOCX) [file pcbi.1012036.s025.docx]

*Table 9:*

| *Vth (mV)* | *E* | *PV* | *SST* | *VIP* |
| --- | --- | --- | --- | --- |
| *L1* |  |  |  | *-40.20* |
| *L2/3* | *-40.53* | *-56.32* | *-39.95* | *-41.34* |
| *L4* | *-47.63* | *-44.23* | *-44.07* | *-40.89* |
| *L5* | *-40.55* | *-51.2* | *-47.38* | *-51.2* |
| *L6* | *-42.31* | *-49.06* | *-37.19* | *-44.81* |
