# Supplementary material for: Cell-type-specific firing patterns in a V1 cortical column model depend on feedforward and feedback-driven states
Source: PLoS Comput Biol. 2025 Apr 23;21(4):e1012036. doi: 10.1371/journal.pcbi.1012036 (PMC12017539; doi:10.1371/journal.pcbi.1012036)
Supplement: S10 Table — (DOCX) [file pcbi.1012036.s026.docx]

*Table 10:*

| *Type of connection* | *Value of sigma* | *Correction factor* |
| --- | --- | --- |
| *E🡪 E* | *114 um* | *0.716* |
| *E🡪 PV* | *92 um* | *0.6244* |
| *E🡪 SST, VIP* | *103 um* | *0.6742* |
| *VIP🡪 E* | *103 um* | *0.6742* |
| *PV🡪 E* | *95 um* | *0.6388* |
| *SST🡪 E* | *85 um* | *0.589* |
| *PV🡪 PV,VIP,SST* | *120 um* | *0.7371* |
